# Chronogram: an R package for data curation and analysis of infection and vaccination cohort studies

**DOI:** 10.1093/bioadv/vbae146

**Published:** 2024-09-27

**Authors:** David Greenwood, Marianne Shawe-Taylor, Hermaleigh Townsley, Joshua Gahir, Nikita Sahadeo, Yakubu Alhassan, Charlotte Chaloner, Oliver Galgut, Gavin Kelly, David L V Bauer, Emma C Wall, Mary Y Wu, Edward J Carr

**Affiliations:** The Francis Crick Institute, London, NW1 1AT, United Kingdom; The Francis Crick Institute, London, NW1 1AT, United Kingdom; National Institute for Health Research (NIHR) University College London Hospitals (UCLH) Biomedical Research Centre and NIHR UCLH Clinical Research Facility, London, W1T 7HA, United Kingdom; The Francis Crick Institute, London, NW1 1AT, United Kingdom; National Institute for Health Research (NIHR) University College London Hospitals (UCLH) Biomedical Research Centre and NIHR UCLH Clinical Research Facility, London, W1T 7HA, United Kingdom; The Francis Crick Institute, London, NW1 1AT, United Kingdom; National Institute for Health Research (NIHR) University College London Hospitals (UCLH) Biomedical Research Centre and NIHR UCLH Clinical Research Facility, London, W1T 7HA, United Kingdom; Department of Preclinical Sciences, Faculty of Medical Sciences, The University of the West Indies, St. Augustine, Republic of Trinidad and Tobago; Department of Biostatistics, School of Public Health, University of Ghana, Accra, Ghana; Yemaachi Biotech, Accra, Ghana; The Francis Crick Institute, London, NW1 1AT, United Kingdom; UCL Centre for Kidney and Bladder Health, Division of Medicine, Royal Free Hospital, London, NW3 2PF, United Kingdom; Clinical Immunology Service, School of Infection, Inflammation and Immunology, College of Medicine and Health, University of Birmingham, Birmingham, B15 2TT, United Kingdom; The Francis Crick Institute, London, NW1 1AT, United Kingdom; The Francis Crick Institute, London, NW1 1AT, United Kingdom; Genotype-to-Phenotype UK National Virology Consortium (G2P-UK), United Kingdom; The Francis Crick Institute, London, NW1 1AT, United Kingdom; National Institute for Health Research (NIHR) University College London Hospitals (UCLH) Biomedical Research Centre and NIHR UCLH Clinical Research Facility, London, W1T 7HA, United Kingdom; Research Department of Infection, Division of Infection and Immunity, UCL, London, WC1E 6BT, United Kingdom; COVID Surveillance Unit, The Francis Crick Institute, London, NW1 1AT, United Kingdom; The Francis Crick Institute, London, NW1 1AT, United Kingdom; UCL Centre for Kidney and Bladder Health, Division of Medicine, Royal Free Hospital, London, NW3 2PF, United Kingdom

## Abstract

**Motivation:**

Observational cohort studies that track vaccine and infection responses offer real-world data to inform pandemic policy. Translating biological hypotheses, such as whether different patterns of accumulated antigenic exposures confer differing antibody responses, into analysis code can be onerous, particularly when source data is dis-aggregated.

**Results:**

The R package chronogram introduces the class chronogram, where metadata is seamlessly aggregated with sparse infection episode, clinical and laboratory data. Each experimental modality is added sequentially, allowing the incorporation of new data, such as specialized time-consuming research assays, or their downstream analyses. Source data can be any rectangular data format, including database tables (such as structured query language databases). This supports annotations that aggregate data types/sources, for example, combining symptoms, molecular testing, and sequencing of one or more infectious episodes in a pathogen-agnostic manner. Chronogram arranges observational data to allow the translation of biological hypotheses into their corresponding code via a shared vocabulary.

**Availability and implementation:**

Chronogram is implemented R and available under an MIT licence at: https://www.github.com/FrancisCrickInstitute/chronogram**;** a user manual is available at: https://franciscrickinstitute.github.io/chronogram/

## 1 Introduction

During an infectious disease outbreak or pandemic, every individual accumulates their own history of antigenic exposures. Prior antigenic exposures shape that individual’s future responses to yet-to-be encountered vaccines and infections (Krammer 2019). Fully annotating these exposures requires date-aware aggregation of several data sources. For example, an infection episode may have symptom diaries, and results from rapid antigen tests, PCRs and/or pathogen sequencing. The interpretation of these infection data can also change over time, such as the influenza subtype indicated by a positive influenza A PCR from one season to the next.

Frequently, human studies store clinical data in secure database solutions such as REDCap (Harris *et al.* 2009, 2019), with research results in laboratory information management systems (LIMS). We propose the chronogram package as a downstream data wrangling and analysis tool with pseudo-anonymized data exported from REDCap and LIMS (or equivalent storage systems).

The R ecosystem already contains a number of packages that are relevant to data carpentry and visualization. The data.table package (Dowle and Srinivasan 2023) offers, in principle, a chronogram-equivalent data structure using keys and indices. The tidyverse ethos (Wickham *et al.* 2019) aligns with our objective to provide a shared vocabulary to facilitate a conversation between humans—clinicians, laboratory scientists, data scientists, and policy makers—and a computer about these data. Whilst the tstibble (time series tibble) package (Wang *et al.* 2020) offers a similar indexed and keyed tibble, we opted to extend the basic tibble rather than increase dependencies. As we add more features, and study follow-up continues (adding *rows*), we continue to evaluate whether data.table (perhaps via dtplyr (Wickham *et al.* 2022)) offers meaningful speed or memory advantages.

Since 2020, we found that we frequently re-constructed rectangular data objects combining metadata with date-indexed experimental data. Between different studies, we needed similar chunks of code to annotate these objects. We wanted our aggregations and annotations to be comparable across parallel studies, and between analysts. In general use, a chronogram is a phrase where letters can be dually interpreted as numbers (often Roman numerals in inscriptions). We use the name chronogram as it captures the time-writing used in the data structure, and as date-aware analysis offers additional, hidden insight. Here, we introduce the chronogram package which allows the date-aware rehearsal of each individual’s antigenic exposures over the course of a pandemic, with a row for each *potential* observation for each individual (a calendar date) and a uniform number of *rows* (days) per participant. Chronogram provides functions to assemble, annotate and sub-cohort datasets to evaluate, for example, the subsequent effects of prior infection with given variants on the next vaccination.

## 2 Methods

The power of chronogram is its rapid, reproducible aggregation of data, and annotation functions, assembling a single data object ready for tidyverse-based, flexible sub-cohorting. Chronogram contains >100 unit tests to ensure consistent assembly, annotation and sub-cohorting. Prospective, or new, chronogram users may wish to review the Section 3 first. Now, we provide a brief step-wise explanation of how to move from source data to a finished chronogram. Further documentation, including a quickstart guide, is available at https://franciscrickinstitute.github.io/chronogram/.

### 2.1 Chronogram assembly

**Figure 1.**
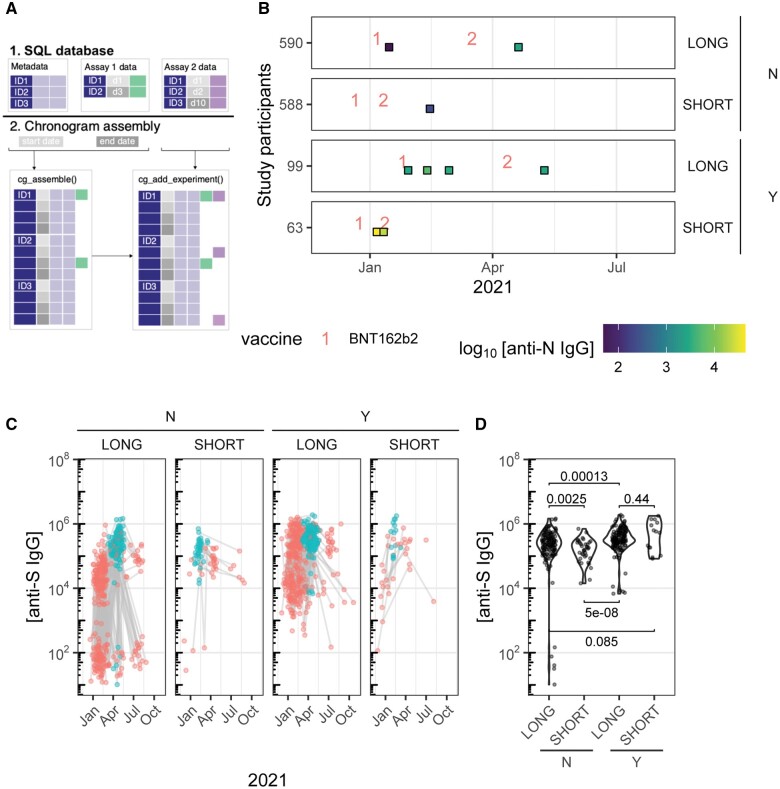
The chronogram package. (A) Schematic illustrating the construction of a chronogram object from tabular data such as SQL database tables. (B) Swimmers plot (cg_plot_metadata()) of four exemplar individuals from the Payne dataset (Payne *et al.* 2021b), first reported elsewhere (Payne *et al.* 2021a). An individual from each group of long (10–12 week) or short (3–4 week) intervals between first and second doses, with doses labelled as 1 or 2; and infection prior to first doses are shown (Y/N). Each venepuncture visit is shown as a square, shaded by the corresponding anti-nucleocapsid IgG (anti-N IgG). (C) Trajectories of anti-SARS-CoV-2 Spike (anti-S) IgG concentrations from the Payne dataset for the four groups described in (B) (cg_plot()). The 28–35 days post second dose results are plotted in blue, the rest of the dataset in red. Sequential samples from the same participant are linked by a grey line. (D) Boxplots of anti-S IgG concentrations responses at 28–35 days after second dose (filter, window, select approach). Statistical comparisons used unpaired two-tailed Wilcoxon tests.

A chronogram is constructed using cg_assemble(), which takes four inputs: start date, end date, a tibble of metadata, and an optional list of tibbles of experiment data (Fig. 1A). Briefly, cg_assemble() generates a row for each individual for each date, and joins metadata and experimental data. cg_assemble() checks input data for duplication and that the resulting chronogram returns only 1 row for each {calendar date, participant ID} combination—the key requirement of our approach. The returned chronogram is a sub-class of tibble (cg_tbl), with attribute slots that store the user-declared column names for calendar date and participant ID, and a slot that lists the metadata columns. Storing these as attributes makes them automatically available to other chronogram functions. With cg_add_experiment(), experiment data can be added later. Vignettes, *vignette(“assembly”, package=“chronogram”), vignette(“SQL_assembly”, package=“chronogram) & vignette(“chronogram_class”, package=“chronogram”)* provide extra details.

### 2.2 Chronogram annotation

We provide a series of functions to annotate chronograms, including:

• Infection episode identification and summarizing
  • cg_annotate_episodes_find() searches backwards and forwards in time to aggregate tests and symptom diaries into distinct episodes of infection. It is common that symptoms and confirmatory testing and viral sequencing are a few days apart. The user provides a vector of columns to scan and a vector of strings to match for each column.
  • cg_annotate_episodes_find_seroconversion() identifies individuals who have seroconverted, indicative of infection. For example, seroconversion to hepatitis B core antigen is suggestive of infection. As the window for exposure (between blood tests) is often much larger than episodes found with cg_annotate_episodes_find(), these functions are kept distinct.
  • cg_annotate_episodes_fill() takes user-specified columns (for example viral sequencing) and adds an additional column where this data has been “filled” over all days of the episode. This allows slicing of the first (or last) row of each episode whilst retaining the desired viral sequencing result. For example, annotating an entire flu episode with A/California/07/2009(H1N1).
• cumulative counting of number of vaccinations, infections, or exposures (vaccinations + infections)
  • cg_annotate_episodes_count()
  • cg_annotate_vaccines_count()
  • cg_annotate_exposures_count()
  • antigenic histories (the sequence of cumulative exposures):
  • cg_annotate_antigenic_history()

These annotations build upon each other: their order of execution matters. We provide vignettes, *vignette(“annotate_vaccines”, package=“chronogram”), vignette(“annotate_episodes”, package=“chronogram”) & vignette(“annotate_exposures”, package=“chronogram”)* to explain their sequential use, and how they can be adapted to studies with several pathogens.

### 2.3 Chronogram Sub-cohorting

Sub-cohorting is the beginning of biological hypothesis testing. Consider comparing antibody responses to a pathogen after four vaccinations and no infection episodes. Sub-cohorting chronograms occur in three stages: *Filter, window*, then *select.*

First, *filter* a chronogram to relevant individuals (for example those with four vaccine exposures, zero infection exposures, and have not seroconverted). Any dplyr filter() translation of this will work. Second, *window* to dates before or after a specific reference event (such as before a vaccination), with cg_window_by_metadata(). Third, *select* a single visit per individual (to avoid retaining >1 result per individual), with cg_select_visit().

The *filter, window, select* process is generalisable, and further windowing functions are provided (cg_window_by_episode(), cg_window_by_visit()). In this example of four doses, we have returned two sub-cohorted chronograms: One before the fourth dose and a second after the fourth dose. The user may wish to combine these for downstream plotting and statistical testing with *dplyr::bind_rows()* after adding a column such as *cohort = {pre dose 4, post dose 4}*.

### 2.4 Chronogram analysis

There are many possible downstream analyses. We provide examples both here (Section 3) and a vignette of approaches to interface chronogram objects with common statistical tests, *vignette(“stats”, package=“chronogram”).*

### 2.5 Chronogram utilities

We provide print(), glimpse(), and summary() implementations for cg_tbl, alongside cg_save() and cg_load() functions. These reduce file size by saving a single row of each participants’ metadata, and load a re-assembled cg_tbl.

## 3 Results and discussion

We illustrate chronogram using a demonstration SQL database of 587 healthcare workers based on the publicly available data to support Payne *et al.* (2021a) shared under a CC BY 4.0 licence (Payne *et al.* 2021b), with the following changes: (i) we have added a random integer between 1 and 28 (DD-) before the publicly released MM-YYYY dates of first dose, and (ii) we have re-calculated other dates using our fictional first dose dates and the provided intervals in days. Whilst not all source data is included, no other changes have been made.

Payne *et al.* (2021a) studied SARS-CoV-2 vaccine responses in BNT162b2 (Pfizer-BioNTech) recipients with differing intervals between the two doses (“short” or “long”), and with and without prior infection. Payne *et al.* (2021a) conducted their study as the UK deviated from the dosing schedule used in the BNT162b2 phase 3 clinical trial (Polack *et al.* 2020): changing from a 3–4 week interval (“short”) to a 10–12 week interval (“long”) between doses 1 and 2. Using a suite of immunological assays, Payne *et al.* showed higher peak responses in the “long” interval (Payne *et al.* 2021a). The peak was larger still in individuals who had prior infection, and therefore three exposures to Spike (two vaccines, one infection). The public Mendeley dataset reflects the kind of experimental data that would become available early during a future pandemic (Payne *et al.* 2021b). Here, we use chronogram to aggregate and explore the dataset.

First, we assemble a chronogram (Fig. 1A—full details in the SQL assembly vignette). Relevant SQL tables are imported with dbplyr::collect(), formatting checked (in particular dates), and subsequently provided to cg_assemble() and cg_add_experiment(). Next, we can illustrate the four important groups: previously uninfected (N) or infected (Y); and long and short dosing intervals (cg_plot_meta(), Fig. 1B). For these four exemplar participants, we find that previously infected individuals have anti-nucleocapsid IgG antibodies throughout (participants 99 and 63) and one participant (participant 590) seroconverts to nucleocapsid during the study. Seroconversion to nucleocapsid strongly suggests an infection episode, as nucleocapsid is encountered only in intact virus (infection) and not vaccine. Thus, nucleocapsid seroconversion by participant 590 between their first and second study visits suggests a SARS-CoV-2 infection between those two visits. Next, we plot the anti-Spike binding antibody titers over time (cg_plot(), Fig. 1C), faceting based on dose interval (long 10–12 weeks; or short 3–4 weeks) and on prior infection status. Payne *et al.* (2021a) compared the day ∼28 [21–35 days] responses after second doses in short- and long-interval groups (samples shaded in blue in Fig. 1C). We repeat that analysis, additionally stratified by prior infection (Fig. 1D). Accepting that our demonstration dataset has incorrect dates (DD- randomization), this plot is reminiscent of the formal result that long interval dosing achieved higher peak antibody responses than short interval dosing, and further suggests this effect is restricted to the group without prior infection. Being date-aware, chronogram highlights the differences between calendar time, and +28 days after a dose (Fig. 1C), and this has implications for the interpretation of these data for policy.

The chronogram package facilitates the incorporation of exposure events with laboratory data on a per-individual basis. Chronogram eases the translation between biology and code, democratizing data analysis of cohort studies, for both current and future pandemics. The package is pathogen-agnostic and can support several pathogens, such as SARS-CoV-2, influenza and respiratory syncytial virus, within a single study, and meta-analyses of several studies.

## Data Availability

The data underlying this article are provided in the chronogram package https://www.github.com/FrancisCrickInstitute/chronogram. The dataset was derived from sources in the public domain: Mendeley Data, https://dx.doi.org/10.17632/fyp26zjgmj.1; Payne *et al.* 2021ahttps://dx.doi.org/10.1016/j.cell.2021.10.011.
